# New Directions in Global Health: How Sweden Can Advance Healthier Populations

**DOI:** 10.34172/ijhpm.2021.99

**Published:** 2021-09-08

**Authors:** Simon Holmesson, Robert Marten, Jesper Sundewall, Stefan Swartling Peterson, Ingrid Petersson, Ole-Petter Ottersen

**Affiliations:** ^1^Swedish Cancer Society, Stockholm, Sweden.; ^2^Alliance for Health Policy and Systems Research, World Health Organization (WHO), Geneva, Switzerland.; ^3^Department of Public Health Sciences, Karolinska Institutet, Solna, Sweden.; ^4^Karolinska Institutet, Stockholm, Sweden.; ^5^Formas, Swedish Research Council, Stockholm, Sweden.

 Just before the coronavirus disease 2019 (COVID-19) pandemic swept across the world in March 2020, the Government of Sweden convened a meeting in Wilton Park to support efforts to create “Healthier Populations.”^1^ Since this meeting, the pandemic has disrupted countries’ health systems and underscored the urgent need to focus on the broader determinants of health. Sweden is learning important lessons from its own challenges in the pandemic. Sweden is also contributing to COVAX and in 2019 provided $717 million for development assistance for health making it one of the top 10 government donors of development assistance for health.^2^ According to the Sustainable Development Solutions Network’s SDG (Sustainable Development Goal) Index, Sweden is the most sustainable country in the world; in 2016, the government stated its intention, “for Sweden to be a leader in the implementation of the 2030 Agenda.” In fact, in Sweden, as recently argued by scholars, health “is framed to highlight health as inextricably tied to societal inequalities.”^3^ Building on this to help advance the “Healthier Populations” agenda, Sweden can contribute by: (1) using the pandemic to bring a systems approach to achieve universal health coverage and address the broader determinants of health; (2); supporting global health governance reform aligned with the SDGs and (3); helping to address the connections between climate change and health.

 The COVID-19 pandemic crisis continues to transform people’s health as well as economies and societies. It is widening disparities and creating uncertainty.^4^ Yet, the current situation also presents an opportunity. It requires taking a systems approach to building stronger, more resilient health systems which can both handle a pandemic and continue providing essential services as part of efforts to move towards universal health coverage. Building on the ongoing COVID-19 experience, countries like Sweden, can help support national health systems improve preparedness to address both pandemics and the broader social, commercial, and environmental determinants of health. This approach would then not only tackle coming pandemics but also ongoing health challenges like non-communicable diseases (NCDs).^5^ Sweden already supports World Health Organization (WHO) to develop its work on Healthier Populations, and recently announced support to the NCD Alliance.^6^ In the future, Sweden could amplify this support by convening these different partners to identify and explore opportunities to apply systems thinking and focus on the broader determinants of health enabling healthier populations.

 Sweden played a critical role in the process to conceptualize SDG 3 to ensure healthy lives and promote well-being for all at all ages; Sweden could now play a similar role in reforming and re-aligning global health governance to focus on SDG3.^7^ The ongoing COVID-19 pandemic puts the SDGs centre stage and shows how current structures and institutions need to transition from the Millennium Development Goals to the wider, more holistic, SDG agenda.^8^ In fact, the pandemic may be used to increase the focus on the multisectoral determinants of health, and the need to see pandemic preparedness in its broader systems context. This also requires systems changes across social, environmental, commercial and political determinants of sustainable health for people and planet, what has been sometimes called a “universal preparedness for health.”^9^ Sweden has already detailed how international institutions could better support the SDG-agenda in its Agenda 2030 implementation report.^10^ The next step is to operationalize this by supporting global institutions to consider the connections between the rapidly rising prevalence of NCDs, the ongoing pandemic and the climate crisis. Understanding these links requires greater investment in local capacities for health policy research, particularly in low- and middle-income countries. As an influential leader in global health, Sweden could support institutions established during the Millennium Development Goal (MDG)-era (like the Global Fund, GAVI and the Joint United Nations Programme on HIV/AIDS) to shift towards creating healthier populations.

 As one of the strongest financial contributors to the Green Climate Fund, Sweden can also highlight and leverage the interlinkages between SDG 3 and climate change.^11^ The health gains of climate action range from improved air quality and more sustainable food systems to urban transport systems which facilitate walking and cycling and bringing health gains from increased physical activity. Meeting the Paris Climate goals could result in health gains twice as large as the costs of the mitigation measures from improved air quality alone and also provide positive economic benefits.^12^ Public concern over the health impacts of climate changes increases public acceptance both globally and domestically in accelerating the work on climate and health fulfilling the SDGs. Sweden has already started supporting the EAT Foundation to accelerate the transformation of the food system in anticipation of the upcoming United Nations (UN) Food System Summit.Next steps could include public health and climate justice considerations in the UN climate negotiations leading up to COP26 in 2021 and raising more awareness of the strong link between public health and climate to support healthier populations.

 Sweden’s approach on healthy population should, just like its feminist policy, aim to continually raise the question on how to create societies for healthier populations.^13^ With the far-reaching implications of the COVID-19 pandemic, many countries are looking for ways to build back better and greener. Sweden should take the lead and fulfil its ambitions for a healthier population by expanding its current efforts to advance a systems approach, support refreshed global governance and address the links between climate and the healthier population agenda.

## Ethical issues

 Not applicable.

## Competing interests

 Authors declare that they have no competing interests.

## Authors’ contributions

 SH led on the drafting of this piece with substantial inputs and guidance from RM in conceptualizing and writing. JS, SWP, IP, and OPO provided feedback and inputs to finalize the draft.
